# Chimeric O1K foot-and-mouth disease virus with SAT2 outer capsid as an FMD vaccine candidate

**DOI:** 10.1038/s41598-018-31856-x

**Published:** 2018-09-12

**Authors:** Abhay Kotecha, Eva Perez-Martin, Yongjie Harvey, Fuquan Zhang, Serban L Ilca, Elizabeth E. Fry, Ben Jackson, Francois Maree, Katherine Scott, Corey W. Hecksel, Michiel M. Harmsen, Valérie Mioulet, Britta Wood, Nick Juleff, David I. Stuart, Bryan Charleston, Julian Seago

**Affiliations:** 1 The Pirbright Institute, Woking, Surrey, GU24 0NF United Kingdom; 2 Transboundary Animal Disease Programme, ARC-Onderstepoort Veterinary Institute, Private Bag X05, Onderstepoort, 0110 South Africa; 3 Division of Structural Biology, Wellcome Trust Centre for Human Genetics, University of Oxford, Oxford, OX3 7BN United Kingdom; 4 Wageningen Bioveterinary Research, Division Virology, P.O. Box 65, 8200 AB Lelystad, The Netherlands; 5 Diamond Light Source, Harwell Science and Innovation Campus, Didcot, OX11 0DE UK

## Abstract

Foot-and-mouth disease virus (FMDV) is highly contagious and infects cloven-hoofed domestic livestock leading to foot-and-mouth disease (FMD). FMD outbreaks have severe economic impact due to production losses and associated control measures. FMDV is found as seven distinct serotypes, but there are numerous subtypes within each serotype, and effective vaccines must match the subtypes circulating in the field. In addition, the O and Southern African Territories (SAT) serotypes, are relatively more thermolabile and their viral capsids readily dissociate into non-immunogenic pentameric subunits, which can compromise the effectiveness of FMD vaccines. Here we report the construction of a chimeric clone between the SAT2 and O serotypes, designed to have SAT2 antigenicity. Characterisation of the chimeric virus showed growth kinetics equal to that of the wild type SAT2 virus with better thermostability, attributable to changes in the VP4 structural protein. Sequence and structural analyses confirmed that no changes from SAT2 were present elsewhere in the capsid as a consequence of the VP4 changes. Following exposure to an elevated temperature the thermostable SAT2-O1K chimera induced higher neutralizing-antibody titres in comparison to wild type SAT2 virus.

## Introduction

Foot-and-mouth disease virus (FMDV) infects cloven-hoofed animals, including domestic livestock such as cattle, pigs and sheep, to cause foot-and-mouth disease (FMD). FMD is enzootic in Africa, Asia and South America. The disease is highly contagious and outbreaks severely impact the economy through the loss of production, trade and tourism in affected regions and present a constant threat for FMD-free countries. FMDV is a member of the *Picornaviridae* family and exists as seven distinct serotypes, namely A, O, C, Asia 1 and Southern African Territories (SAT) 1, 2 and 3, with numerous subtypes within each serotype^1^.

The FMDV genome and structural composition of the FMDV capsid have been well documented; infectious FMDV particles have non-enveloped icosahedral protein capsids containing a single-stranded, positive-sense RNA genome approximately 8500 nt in length^2,3^. IRES-mediated translation of the FMDV genome yields a single polyprotein that is processed proteolytically to generate intermediate precursors and mature proteins. During translation an intra-ribosomal self-processing event occurs at the C terminus of 2A, separating the region containing the capsid proteins (P1) and non-structural 2A from the rest of the polyprotein. P1–2A is subsequently processed by the 3C protease to generate VP0, VP3, VP1 and 2A. A single molecule each of VP0, VP1 and VP3 assemble to form a protomer. Five protomers assemble to form a pentamer, and 12 pentamers assemble to generate an intact capsid enclosing the viral genome, with VP0 cleavage occurring usually after genome encapsidation^4^. VP4 is entirely internal to the capsid, sandwiched between the outer capsid proteins (VP1–VP3) and the genome, and is usually lost from picornavirus capsids during the uncoating process^5^.

One of the main challenges facing pathogen eradication and control campaigns is the lack of suitable and cost-effective vaccines. The antigen compositions of vaccines destined for FMD-endemic regions are often not tailored for their specific needs; vaccines produced from one subtype may not fully protect against circulating disparate subtypes^6^. Of particular note is the genetic and antigenic variability exhibited by the SAT serotypes of FMDV, driven by the independent evolution of these viruses in different geographic regions^7^. The characterisation and adaptation to cultured cells of such circulating strains, in order to facilitate their use for vaccine production, is both time consuming and technically challenging. Another factor is the stability of the SAT serotypes, which are amongst the most temperature labile^8^. One approach to overcome such obstacles involves the construction of infectious clones that can be genetically manipulated and the subsequent production of recombinant viruses.

Here we report the construction and characterisation of chimeric SAT2 viruses encoding the outer capsid proteins of SAT2 in the genetic background of O1Kaufbeuren (O1K). We show the SAT2 chimeras are more thermostable than the respective wild type viruses and have identified the residues predominantly responsible for the observed thermostability. Sequence and electron cryo-microscopy (cryo-EM) analyses of the chimeric viruses confirmed that no other changes were present and the native antigenic structure was conserved. We show such thermostable SAT2 viruses can induce improved neutralizing-antibody responses following the exposure of vaccine antigen to an elevated temperature.

## Results

### Construction of chimeric SAT2/O1K infectious clone

**Figure 1:**
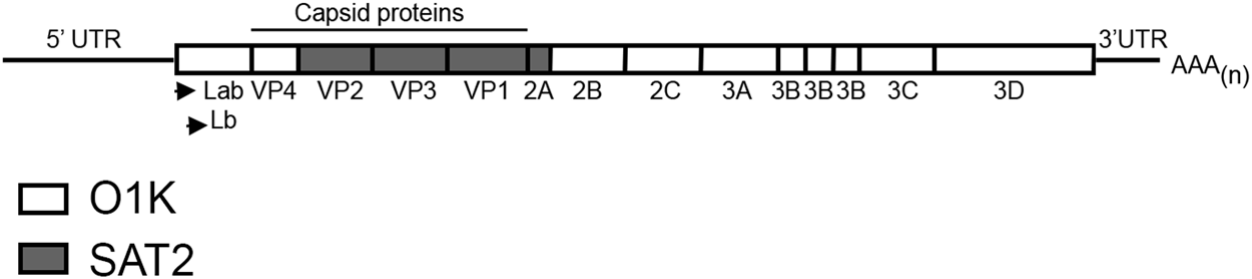
Schematic representation of the chimeric SAT2/O recombinant FMDV genome, encoding the VP4 inner-capsid structural protein and Lpro (Lab and Lb forms), 2B, 2C, 3A, 3B, 3C and 3D non-structural proteins of FMDV O1K (white boxes) in combination with the outer capsid proteins (VP2, VP3 and VP1) and the non-structural 2A product of SAT2 Zim/7/83 (grey boxes). The 5′UTR, 3′ UTR and poly (A) tail are also depicted.

We have previously used reverse genetics to construct chimeric infectious clones of FMDV O serotype; these encoded the VP4 inner structural protein and almost all the non-structural proteins (NSPs) (Lpro, 2B, 2C, 3A, 3B, 3C and 3D) of FMDV O1K in combination with the outer capsid proteins (VP2, VP3 and VP1) and the non-structural 2A product of either the O1Manisa (O1M) or OUKG subtypes^9–12^. To determine if the SAT2 structural proteins can be effectively processed by O serotype NSPs, we used a similar cloning strategy to generate a SAT2/O1Kaufbeuren (O1K) chimeric clone encoding the outer capsid proteins and the non-structural 2A product of SAT2 ZIM/7/83, with NSPs and VP4 from O1K^13^ (Fig. 1). Indeed, RNA transcribed from the SAT2/O1K clone and electroporated into BHK-21 cells gave rise to infectious FMDV (referred to as SAT2/O).

### Chimeric SAT2 infectious clone has an improved thermostability

**Figure 2:**
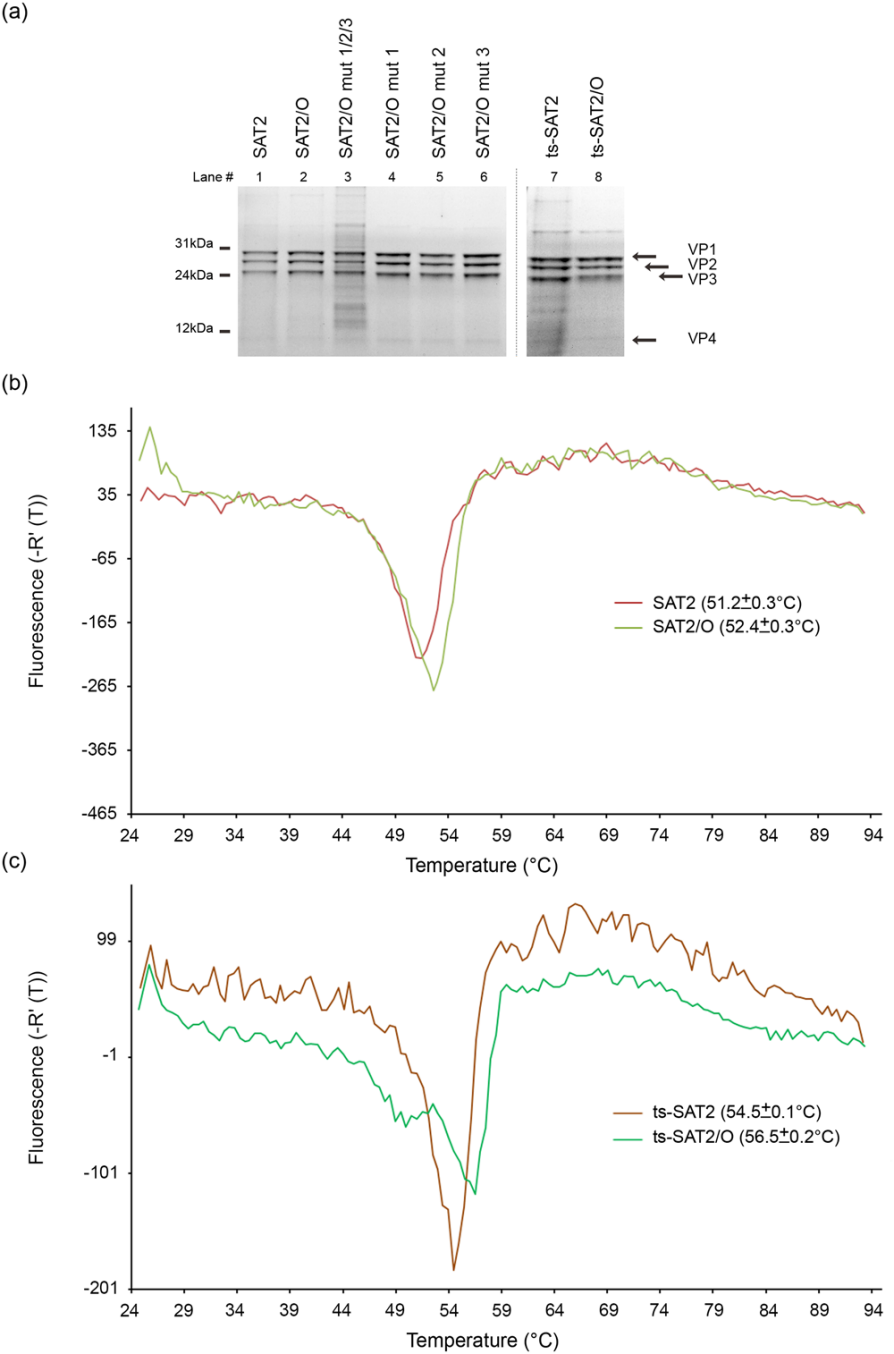
Thermostability analysis of chimeric SAT2/O recombinant FMDVS. (**a**) SDS-PAGE gel analysis of purified SAT viruses used to perform thermofluor assays. Lanes containing SAT2, SAT2/O, SAT2/O mut1/2/3, SAT2/O mut1, SAT2/O mut2, SAT2/O mut3, ts-SAT2 and ts-SAT2/O viruses are indicated. Arrows specify bands corresponding to the VP1–4 structural proteins. (**b**) Thermofluor analysis to determine capsid stability of wild type SAT2 and SAT2/O viruses. (**c**) Thermofluor analysis to determine capsid stability of ts-SAT2 and ts-SAT2/O viruses. The respective temperatures of genome release, and hence capsid disassembly, for three independent experiments are shown in brackets.

SAT serotype capsids are particularly thermally unstable and readily dissociate into pentameric subunits^8^. To compare the thermostabilities of the wild type SAT2 and chimeric SAT2/O capsids the viruses were purified by sucrose gradient centrifugation^14^, analysed by SDS-PAGE and Coomassie blue staining to visualise components of the viral capsid and assess purity (Fig. 2a, lanes 1 and 2), and then used to perform thermofluor-based assays (Fig. 2b). The thermofluor assay monitors viral genome release, as an indicator of capsid disassembly, during a slow increase in temperature using a dye sensitive to the presence of ribonucleic acid^15,16^. In repeated thermofluor assays the temperature at which the RNA genome was released (T_R_) was consistently higher for SAT2/O than for wild type SAT2 (T_R_ = 52.4 °C ± 0.3 and 51.2 °C ± 0.3, respectively), suggesting an increase in thermostability of the SAT2/O capsid (Fig. 2b). To confirm this, we utilised a similar pair of recombinant SAT2 viruses, termed ts-SAT2 and ts-SAT2/O. These viruses encoded the same amino acid sequences as the respective SAT2 and SAT2/O viruses, aside from one substitution (VP2 93 Ser to Tyr) conferring an increase in thermostability of the capsid; this mutation has recently been described by Kotecha and colleagues^17^. As expected, thermofluor analysis using purified viruses (Fig. 2a, lanes 7 and 8) confirmed ts-SAT2 was more thermostable (T_R_ = 54.5 °C ± 0.1) than both SAT2/O and the wild type SAT2 virus (Fig. 2c). Surprisingly, a further increase in thermostability was observed for ts-SAT2/O (T_R_ = 56.5 °C ± 0.2) (Fig. 2c).

### O1K VP4 is responsible for the increase in thermostability of chimeric SAT2/O

**Figure 3:**
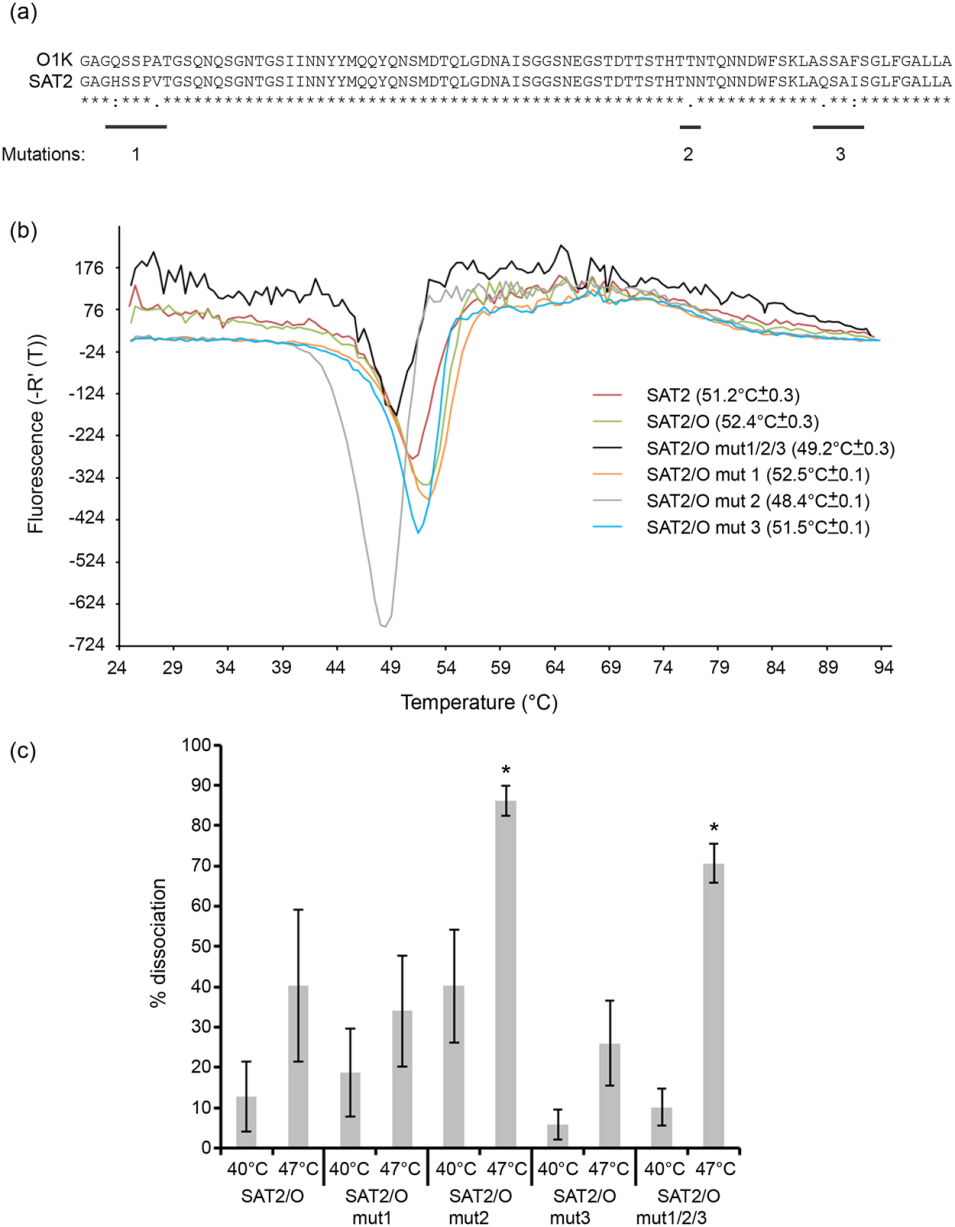
Thermostability analysis of chimeric SAT2/O recombinant FMDVs containing targeted mutations in the VP4 non-structural protein. (**a**) Alignment of the VP4 amino acid sequences of O1Kaufbeuren (O1K) and SAT2 ZIM/7/83 FMDV strains. Residues that are conserved (asterisks), have strongly similar properties (colons) or similar properties (periods) are indicated. (**b**) Thermofluor analysis to determine the capsid stability of wild type SAT2, SAT2/O and SAT2/O viruses containing one (SAT2/O mut 1, SAT2/O mut 2, SAT2/O mut 3) or three (SAT2/O mut 1/2/3) targeted VP4 mutations. The respective temperatures of genome release, and hence capsid disassembly, for three independent experiments are shown in brackets. (**c**) Analysis of capsid stability by double antibody sandwich ELISA using a single-domain llama antibody (M377F VHH) that specifically recognises intact SAT2 FMDV capsid (146S). SAT2/O and SAT2/O viruses containing one (SAT2/O mut 1, SAT2/O mut 2, SAT2/O mut 3) or three targeted (SAT2/O mut 1/2/3) VP4 mutations were incubated at different temperatures (4 °C, 40 °C and 47 °C) for 10 minutes and the respective percent dissociation, as determined by the loss of 146S, was calculated following incubation at each temperature. Error bars indicate standard deviation and asterisks indicate a significant (Tukey’s method; 95% confidence, P < 0.05) increase in dissociation.

We reasoned that the observed increase in thermostability of the chimeric SAT2/O viruses may be mediated by the presence of the O1K VP4 structural protein. Alignment of the respective VP4 amino acid sequences of O1K and SAT2 revealed an identity of 94% and only 5 non-identical residues (Fig. 3a). Site-directed mutagenesis was used to change the O1K VP4 amino acid sequence to that of SAT2 in three sequential rounds of mutation, mutations 1 (VP4 4Q to H and VP4 8A to V), mutation 2 (VP4 60T to N) and mutations 3 (VP4 73S to Q and VP4 76F to I); the resultant infectious recombinant clone was termed SAT2/O mut1/2/3. RNA transcribed from the SAT2/O mut1/2/3 clone and electroporated into BHK-21 cells generated infectious FMDV.

Next, the thermostability of purified SAT2/O mut1/2/3 (Fig. 2a, lane 3) was investigated using thermofluor analysis (Fig. 3b). This virus exhibited a reduced thermostability compared to SAT2/O (T_R_ = 49.2 °C ± 0.3 and 52.4 °C ± 0.3, respectively). Indeed SAT2/O mut1/2/3 was also less thermostabile than the wild type SAT2 virus (T_R_ = 49.2 °C ± 0.3 and 51.2 °C ± 0.3, respectively).

Our next aim was to identify if a specific mutation was responsible for the decrease in thermostability of SAT2/O mut1/2/3. To do this, three further infectious viruses were generated (termed SAT2/O mut1, SAT2/O mut2 and SAT2/O mut3), each of which contained one of the sequential rounds of mutation present in the SAT2/O mut1/2/3 virus. Purified SAT2/O mut1, SAT2/O mut2 and SAT2/O mut3 (Fig. 2a, lanes 4, 5 and 6) were then used for thermofluor analysis alongside the related viruses (SAT2/O mut1/2/3, SAT2/O and wild type SAT2). As Fig. 3b shows, the SAT2/O mut1 virus exhibited comparable thermostability (T_R_ = 52.5 °C ± 0.1) to SAT2/O, and was more stable than the wild type SAT2 virus (T_R_ = 51.2 °C ± 0.3). SAT2/O mut3 showed an intermediate thermostability (T_R_ = 51.5 °C ± 0.1) between SAT2/O and SAT2. However, the thermofluor data for SAT2/O mut2 was of particular interest, as the stability of this virus (T_R_ = 48.4 °C ± 0.1) was even lower than that of SAT2/O mut1/2/3 (T_R_ = 49.2 °C ± 0.3). These results indicate that although the mut3 changes, VP4 73S to Q and VP4 76F to I, may have contributed, the single mut2 reversion to the SAT2 sequence (VP4 60T to N) is sufficient to abolish the stabilising effect achieved by adding O components to produce the SAT2/O chimeric virus. Sequence analysis of the P1 capsid regions of all recombinant SAT2 viruses revealed no additional mutations other than those targeted to VP4 (data not shown).

To corroborate the thermofluor data generated using the mutated SAT2/O viruses we used an ELISA-based technique which uses a single-domain llama antibody (M377F VHH) that specifically recognises intact FMDV capsid (146S)^18^.

Purified samples of the parental SAT2/O virus and the related mutant viruses (SAT2/O mut1/2/3, SAT2/O mut1, SAT2/O mut2 and SAT2/O mut3) were incubated at different temperatures (4 °C,  40 °C or 47 °C) and the dissociation of their respective capsids determined (Fig. 3c and Supplementary Table 1). Indeed, statistical analysis confirmed the SAT2/O mut1/2/3 and SAT2/O mut2 viruses exhibited significantly (Tukey’s method; 95% confidence, P < 0.05) more 146S dissociation at 47 °C in comparison to the parental SAT2/O virus. Specifically, the ELISA analysis showed almost 90% of the SAT2/O mut2 virus had dissociated after incubation at 47 °C, closely correlating with its thermofluor T_R_ (48.4 °C ± 0.1).

To investigate if any of the chimeric SAT2/O viruses showed altered pH stability in comparison to wild type SAT2, thermofluor analysis was performed at various pH values below or above pH7.4 (pH6, 6.3, 6.5, 6.8, 7, 8, 8.5 and 9) (Supplementary Table 2). The highest stability for all the viruses was observed at pH8. Interestingly, in comparison to both wild type SAT2 and SAT2/O, the SAT2/O mut1 virus exhibited higher T_R_ values over the tested pH range.

**Figure 4:**
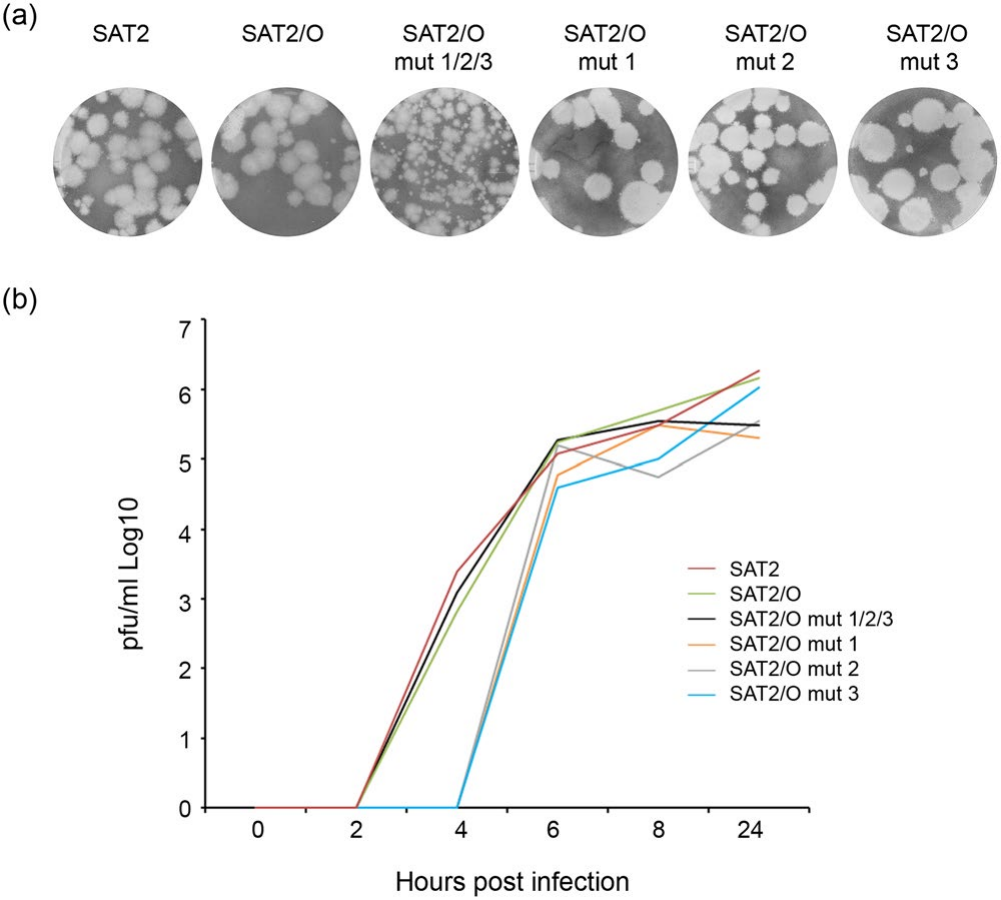
Growth properties of chimeric SAT2/O recombinant FMDVs. (**a**) Plaque morphology of wild type SAT2, SAT2/O and SAT2/O viruses containing one (SAT2/O mut 1, SAT2/O mut 2, SAT2/O mut 3) or three targeted (SAT2/O mut 1/2/3) VP4 mutations. (**b**) Growth analysis comparison of wild type SAT2, SAT2/O, SAT2/O mut 1, SAT2/O mut 2, SAT2/O mut 3 and SAT2/O mut 1/2/3. Goat epithelium cells were infected with the respective virus (0.01 m.o.i.) and samples analysed at 0, 2, 4, 6, 8 and 24 h post-infection. Similar results were obtained from three individual experiments.

To compare growth characteristics of the wild type and recombinant viruses, plaque assays and growth curves were performed. Most of the viruses produced plaques of similar morphology, however, those of SAT2/O mut1/2/3 were comparatively smaller (Fig. 4a). The SAT2, SAT2/O and SAT2O mut1/2/3 viruses showed similar growth kinetics, in comparison, a slightly reduced rate of growth was observed for the remaining viruses (SAT2/O mut1, SAT2/O mut2 and SAT2/O mut3) (Fig. 4b).

### Structure analysis of stabilized capsids

**Figure 5:**
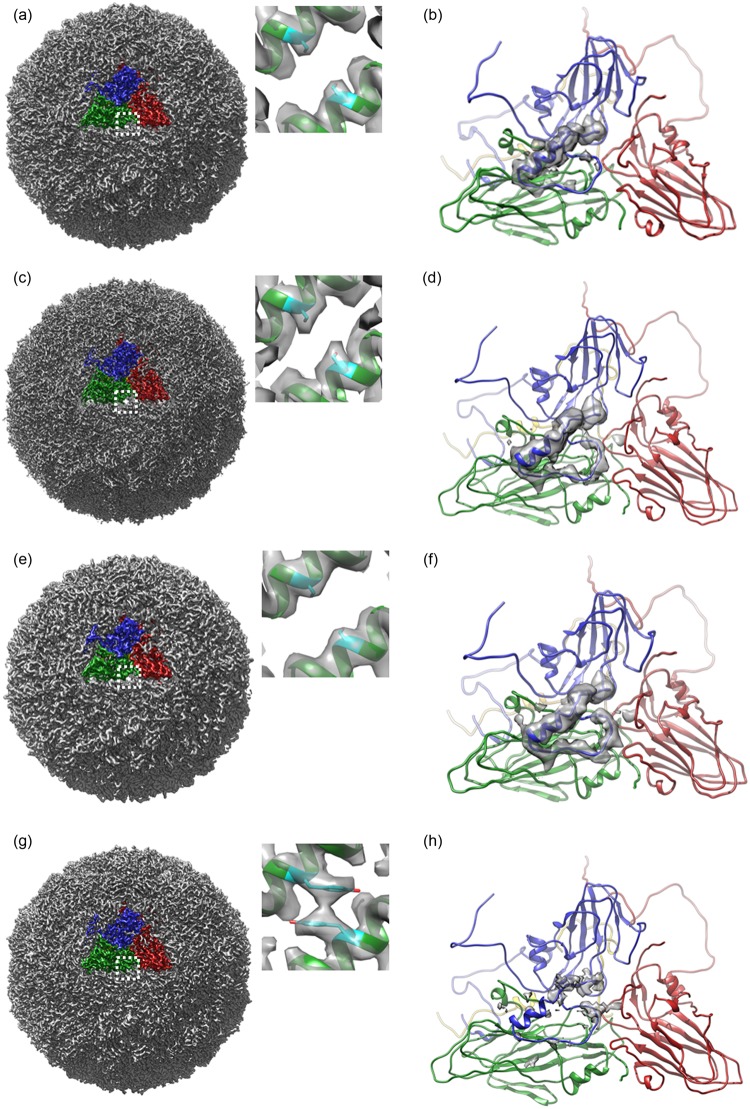
CryoEM maps and atomic models of the chimeric viruses, (**a**) a whole virus map of SAT2 wildtype particle. A single protomer is highlighted and coloured as VP1 – blue, VP2 – green and VP3 – red. The inset shows the zoomed-up density of the VP2 alpha-helix at the icosahedral 2-fold axis with a fitted atomic model. The side chain for VP2 residue 93S is shown in stick representation in cyan. (**b**) Atomic model for SAT2 wildtype. Colouring reflects the different viral proteins, VP1 (blue), VP2 (green), VP3 (red) and VP4 (yellow). The VP1 GH loop is in the down conformation, the associated EM density is shown in grey. (**c**) and (**d**) EM electric potential map and atomic model of SAT2/O, depicted as (**a**) and (**b**) respectively. (**e**) and (**f**) EM electric potential map and associated atomic model of SAT2/O mut2, depicted as (**a**) and (**b**) respectively. (**g**) and (**h**) EM electric potential map and associated atomic model of ts-SAT2/O, depicted as (**a**) and (**b**) respectively. The inset shows the tyrosine sidechain making predicted stacking interactions at the 2-fold axis at position VP2 93. The VP1 GH loop is found to be flexible and hence no density is visible in the EM map.

We determined structures of four capsids by cryo-EM. 8935 particles of SAT2 Zim/7/83, 6606 particles of SAT2/O, 3554 particles of ts-SAT2/O and 8776 particles of SAT2/O mut2 (VP4 60N) yielded structures at 3.5, 2.8, 3.7 and 3.6 Å resolution (Fourier shell correlation of 0.143), respectively (Fig. 5, Supplementary Fig. 1 and Supplementary Table 3). Refinement used strict non-crystallographic symmetry and yielded reliable models (Supplementary Table 3). The external features of the refined atomic models were very similar for these viruses, with the exception that in the ts-SAT2/O structure containing the VP2 93 Y mutation, the VP1 GH loop appeared more disordered, as did the GH loop of VP3. In contrast, the VP1 GH loops of the SAT2/O and SAT2/O mut2 chimeric viruses were visible at approximately 70% occupancy in the ‘down’ conformation, packed against the virus surface^19^. In all the particles, VP4 residues 1–14 and 40–64, were disordered. A MoreRONN plot (Supplementary Fig. 2) of the VP4 sequences for O1K and SAT2 predicts this region to be disordered, as indeed it has been in all the FMDV serotype structures solved to date. It is feasible to suggest that these regions interact with the RNA and that the VP4 T60N mutant affects the particle stability by virtue of its association with RNA.

### Validation of cloning strategy and immunogenicity of chimeric SAT2

To validate the cloning strategy used to produce SAT2/O could be used to generate another chimeric SAT2 virus with increased stability, an infectious copy plasmid of SAT2 ETH 65/09 was constructed. As before, RNA transcribed from the respective clone and electroporated into BHK-21 cells gave rise to an infectious chimeric SAT2 FMDV (referred to as v2-SAT2/O). Purified preparations of v2-SAT2/O, as well as the wild type virus (referred to as v2-SAT2), were then used for thermofluor analysis. In agreement with our above mentioned SAT2/O data, v2-SAT2/O exhibited increased stability in comparison to wild type v2-SAT2 virus (T_R_ = 52.1 °C ± 0.2 and T_R_ = 50.1 °C ± 0.1, respectively).

**Figure 6:**
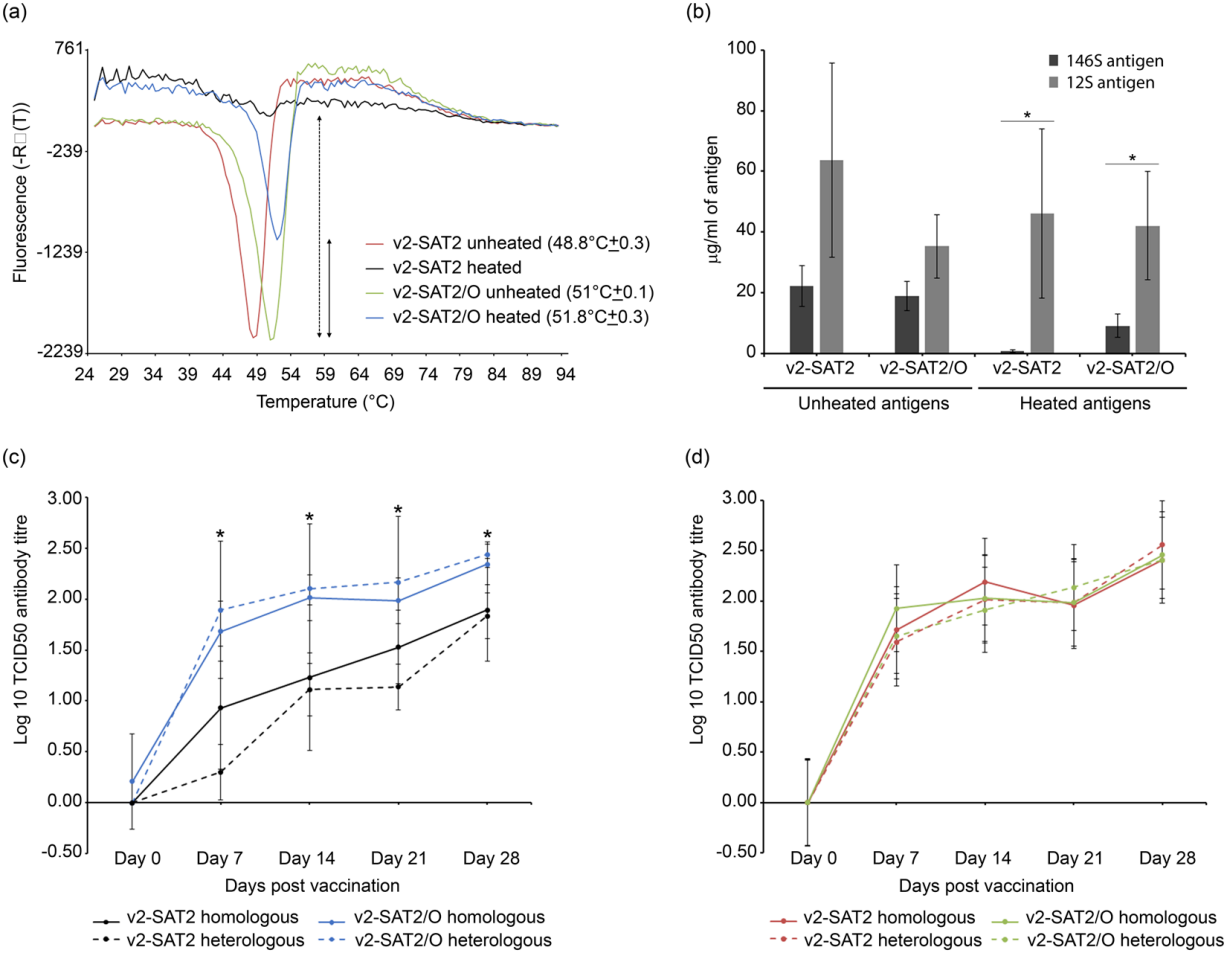
Assessment of vaccine quality and immunogenicity following exposure to an elevated temperature. (**a**) Thermostability analysis of v2-SAT2 and v2-SAT2/O vaccines before (unheated) and after (heated) incubation at 45 °C for 2 hours. The respective temperatures of genome release, and hence capsid disassembly, for three independent experiments are shown in brackets. The reduction in fluorescence signal due to loss of intact capsid (146S) is represented by dashed and non-dashed arrows for heated v2-SAT2 and v2-SAT2/O, respectively. (**b**) Analysis of v2-SAT2 and v2-SAT2/O vaccines before and after incubation at 45 °C for 2 hours by double antibody sandwich ELISA using a single-domain llama antibody (M377F VHH) that specifically recognises 146S. Asterisks indicate a significant decrease (Tukey’s method; 95% confidence, P < 0.05) in the respective 146S:12S vaccine composition following heat treatment. (**c**,**d**) Groups of five calves were vaccinated with the respective vaccine at days 0 and boosted on day 21; blood samples were assayed at 0, 7, 14, 21, and 28 days post vaccination. Homologous and heterologous VNT results are shown. Error bars indicate standard deviation. (**c**) Mean virus neutralizing-antibody titers (VNT (log_10_)) of calves vaccinated with heat-treated (45 °C for 2 hours) v2-SAT2 or v2-SAT2/O vaccines. Asterisks indicate a significant difference (Tukey’s method; 95% confidence, P < 0.05) in neutralising titres between vaccinate groups. (**d**) Mean virus neutralizing-antibody titers (VNT (log_10_)) of calves vaccinated with unheated v2-SAT2 or v2-SAT2/O vaccines.

In contrast to the SAT2/O virus, the v2-SAT2/O virus consistently exhibited a greater increase (2 °C) in thermostability in comparison to the respective wild type virus (SAT2 and v2-SAT2, respectively). This prompted us to use v2-SAT2/O and v2-SAT2 as vaccine antigens and to compare their respective immunogenicity following exposure to an elevated temperature. To do this, we vaccinated calves with inactivated v2-SAT2/O or v2-SAT2 following exposure of equal quantities of intact viral antigens to 45 °C for 2 hours. Control animals were vaccinated with unheated viral antigens maintained at 4 °C. Antigen samples were analysed before and after heat treatment by thermofluor assay, and by ELISA to quantify 12S and 146S levels. Thermofluor analysis showed that although the inactivation process reduced the thermal stability of both viruses, v2-SAT2/O retained a higher thermostability in comparison to v2-SAT2 (Fig. 6a); a larger inverse dissociation peak also indicated the presence of more intact particles in the heated v2-SAT2/O antigen in comparison to the heated v2-SAT2 antigen. Interestingly, the heated v2-SAT2/O antigen exhibited a slightly increased stability in comparison to unheated v2-SAT2/O (51.8 °C ± 0.3 and T_R_ = 51 °C ± 0.1, respectively). Statistical analysis revealed a significant decrease in the respective 146S:12S composition (Tukey’s method; 95% confidence, P < 0.05) of both vaccines following heat treatment. However, in comparison to the presence of approximately 20 μg/ml of 146S in both vaccines prior to heat treatment, the v2-SAT2 and v2-SAT2/O vaccines contained approximately 1 and 9 μg /ml of 146S, respectively, after heat treatment (Fig. 6b).

Serum virus neutralization antibody titers (VNTs) were measured at 0, 7, 14, 21 and 28 days post-vaccination using homologous and heterologous viruses (Fig. 6c,d). In comparison to animals vaccinated with the heated v2-SAT2 vaccine, those vaccinated with heated v2-SAT2/O vaccine generated significantly higher (Tukey’s method; 95% confidence, P < 0.05) mean neutralising antibody titres on days 7, 14, 21 and 28 (Fig. 6c). Furthermore, homologous VNT results showed mean antibody titers >2 log_10_ (considered protective^20^) on day 28 for animals vaccinated with heated v2-SAT2/O but not those vaccinated with heated v2-SAT2 (Fig. 6c). In comparison, mean antibody titers >2 log_10_ were observed for both the unheated v2-SAT2/O and v2-SAT2 vaccines on day 28 (Fig. 6d). In addition, no significant difference was observed between the homologous and heterologous VNTs performed with sera samples from v2-SAT2/O vaccinates, confirming the antigenic conservation between wild type and chimeric SAT2 viruses.

## Discussion

In this study, we constructed an infectious chimeric FMDV clone encoding the external capsid proteins of the unstable SAT2 strain ZIM/7/83 and the VP4 inner structural protein and almost all the NSPs of FMDV O1K. This chimera (SAT2/O) had growth kinetics equal to that of the wild type SAT2 virus, but was incrementally more thermostable. We investigated if the O1K VP4 structural protein was responsible for the increase in thermostability of SAT2/O. Indeed, a series of targeted mutations designed to revert the O1K VP4 protein to that of SAT2 identified C-terminal residues (VP4 60T to N, 73S to Q and 76F to I) that reduced the thermostability of the SAT2/O chimera. Interestingly, two N-terminal mutations (4Q to H and 8 A to V) of O1K VP4 increased the thermostability of the SAT/O capsid when analysed at a range of different pH values (Supplementary Table 2).

A similar infectious clone encoding the external capsid coding region of SAT2 strain, ZIM/7/83, in the genetic background of type A_12_ (termed vSAT2/A12) has been previously described^21^. As reported for all our chimeras, the authors were able to recover viable virus, however, the published vSAT2/A12 chimera had slower growth kinetics and a lower thermostability than the wild type SAT2 virus. In a subsequent publication, Van Rensburg *et al*. made a series of chimeric SAT2/A12 viruses to investigate the underlying factors for the observed properties of vSAT2/A12^13^. The authors concluded that there were incompatibilities between the 5′ region of A12, encompassing the 5′UTR, Lpro and VP4, and the SAT2 genome. The VP4 amino acid sequences of O1K and A12 serotypes share an identity of 98.8% and differ by only one amino acid at the C-terminus of VP4 (76S and 76T, respectively) (data not shown), whether this is the destabilising factor for the observed differences in the respective stabilities of the SAT2/O and SAT2/A12 in comparison to the wild type SAT2 virus remains to be determined.

FMDV is particularly pH and heat labile, its capsid readily dissociates below pH 7, even under cold chain conditions^8,22^. However, with regards to thermostability the seven serotypes of FMDV display significant differences. Doel and Baccarini showed that live viruses of types A and C are more stable than types O and SAT1-3, with SAT viruses being particularly sensitive to temperature^8^. To determine the thermostability of the viruses used in this study two recently reported techniques were used, (i) thermofluor analysis (also termed PaSTRy: Particle STability using Release assaY) which monitors genome release as a consequence of capsid dissociation and (ii) ELISA using a llama single-domain antibody that recognises intact FMDV particles (146S infectious virions or 75S VLPs)^15,16,18,23,24^. In these assays purified virus samples were used to standardise experimental conditions and to ensure samples predominantly consisted of 146S intact virions. An alternative thermofluor-based methodology that monitors protein denaturation, and not the presence of RNA, using a dye sensitive to the presence of hydrophobic residues was not used in this study as in our hands this technique was less sensitive and required larger quantities of purified viruses^16^. Similar findings were reported by Walter and colleagues, who used thermofluor analysis to investigate both genome release and protein denaturation of equine rhinitis A virus, bovine enterovirus type 2 and poliovirus^15^. Likewise, comparable ELISA-based methodologies have been described for the quantification of intact 146S particles in vaccine preparations^25^. The llama antibody, M377F VHH, used in this study was produced as previously reported for an analogous llama antibody (M170 VHH) recognising the intact capsid of FMDV serotype O^18,23,24^. Supplementary Figure 3 shows the specificity of M377F VHH, as heated samples that were no longer recognised by the llama antibody (Fig. 3c) were by polyclonal serum that detects both 146S and 12S (capsid subunit) fractions.

The structural precursor P1–2A is cleaved by FMDV 3 C protease to yield VP0, VP3, VP1 and 2 A. Cleavage of VP0 to VP4 and VP2 has been shown to occur on encapsidation of the viral RNA and also within assembled VLPs^5,26^. In this study, analysis of purified SAT2/O mut2 virus by SDS PAGE and Coomassie staining showed VP0 was cleaved (Fig. 2a, lane 5). In addition, our cryo-EM analyses verified that the SAT2/O mut2 capsids were similar to wild type SAT2 virus. In infectious virions VP1, along with VP2 and VP3 form the external surface of the FMDV capsid, whilst VP4 is localised internally, in close proximity to the viral genome. Studies on other picornaviruses have provided evidence that VP4 is released from the virus in the initial stages of infection, forming multimeric, size-selective membrane pores in order to overcome the host membrane barrier and deliver the viral genome into the cytosol^27–29^. We have shown that a chimeric virus expressing the NSPs of O1K and capsid proteins of SAT2 becomes more stable when VP4 is replaced by that of O1K. It is possible that the predominantly O1K genome of the chimeric SAT2/O virus, either through physical interaction with O1K VP4 and/or as a consequence of its inherent thermodynamic properties, contributed to the observed increase in thermostability. To date, it is not known how VP4 interacts with the viral genome. The FMDV genome *per se* has been shown to influence both pH and thermal stability in FMDV. Curry and co-workers showed that empty capsids and synthetic VLPs of three subtypes of serotype A were more stable by 0.5 pH unit on average than the corresponding virion^26^, suggesting that the viral RNA modulates acid lability in FMDV. In contrast, empty particles were reported to be more sensitive to temperature and readily dissociate^8^. The crystal structure of the empty capsid of type A22 Iraq 24/64 showed that the N-termini of VP1 and the C-terminii of VP4 are less well ordered in the absence of genome, which induces protein folds that enhance capsid stability^30^.

To confirm that the observed increase in thermostability of the chimeric SAT2 viruses could influence the immunogenicity following exposure to an elevated temperature, we heated chimeric and wild type SAT2 inactivated viruses prior to vaccination. The heated chimeric SAT2 vaccine performed markedly better than the heated wild-type SAT2 vaccine, eliciting significantly higher neutralising antibody titres consistent with protection. Our analyses of the vaccinated samples indicate this was facilitated by the comparatively higher levels of 146S antigen present in the thermostable chimeric SAT2 vaccine. Future studies will extend this observation and investigate the duration and breadth of protection that such recombinant SAT2/O vaccines confer to FMDV challenge.

This study is an extension of previous work carried out by the authors and contributes to a continued improvement in the generation of FMDV vaccines^17,31–34^. These studies have identified some of the structural determinants of capsid thermolability and shown that the stability of SAT2 viruses can be improved through predictive modeling^17,31,32^. In addition, they have shown that chimeric SAT2 vaccines can induce protective immune responses^33,34^. The present study has highlighted the feasibility of using capsid switching to generate recombinant FMDVs with improved thermal stability for particularly labile serotypes such as the SATs. In addition, we have shown that the inclusion of a targeted mutation, previously reported to increase capsid stability, led to an accumulative increase in thermal stability^17^. With the rapid advancement of microscopy techniques, it is possible that molecular interactions in the internal environment of the FMDV capsid, specifically those between the RNA genome and the structural components, may soon be determined at a high enough resolution to inform the further optimisation of chimeric FMDVs. In combination with recently reported mutations that facilitate cell culture adaptation and increase the repertoire of cells susceptible to FMDV infection, it will be possible to rapidly produce stable recombinant FMD vaccines that match circulating strains, and perhaps ultimately switch to engineered VLP antigen^35,36^.

## Materials and Methods

The authors confirm that the data supporting the findings of this study are available within the article [and/or] its supplementary materials.

### Construction of chimeric viruses

Infectious FMDV O1K/O UKG35 and tagged FMDV O1K/O1Manisa (O1M) chimeric clones were constructed using reverse genetics as described previously^10,12^. Briefly, cDNA encoding the VP2, VP3, VP1 and 2 A proteins was removed from an existing O1K infectious clone leaving cDNA encoding the Lpro, VP4, 2B, 2C, 3A, 3B, 3C, 3D proteins. The removed cDNA was replaced with the corresponding SAT2 (ZIM 7/83 or ETH 65/09) cDNA from pGEM9zf sub-clones to generate SAT2/O and V2-SAT2/O infectious copy plasmids, respectively. Infectious clones SAT2/O mut 1/2/3, SAT2/O mut1, SAT2/O mut2 and SAT2/O mut3 were generated by targeted point mutation in VP4 using the QuikChange Lightning Site-Directed Mutagenesis Kit (Agilent technologies, UK) according to the manufacturer’s instructions. Construction of infectious copy plasmids and all live virus work was performed at a SAPO4 level of biocontainment.

### Preparation of infectious RNA and electroporation

RNA transcribed from the full length infectious clones made using the MEGAscript® T7 Kit (Invitrogen, UK) was electroporated into BHK-21 cells using a Biorad Gene PulsarTM (two pulses at 0.75 kV and 25 μFD). Cell lysates prepared after 24 hours were used subsequently to passage the viruses on a goat epithelium cell line^37^. Methods involving vertebrate cell samples were performed in accordance with relevant regulations and guidelines and methods were approved by The Pirbright Institute.

### Genome amplification and sequencing

Total RNA was extracted using Trizol reagent (Invitrogen, UK) and the respective region of the viral RNA genome was reverse transcribed and amplified by PCR using a “One-Step RT-PCR Kit” (Qiagen, UK). Sequencing reactions were then performed using an aliquot of the purified PCR product and the BIG Dye Terminator v3.1 cycle sequencing kit (Applied Biosystems, UK). When sequencing was performed to confirm the absence of additional mutations in recombinant SAT2 viruses, purified virus preparations were used as the source of RNA for RT-PCR amplification.

### Virus growth curve and plaque assay

Confluent monolayers of goat epithelium cells were infected with chimeric and wild type SAT2 viruses at the same MOI (0.01). Unabsorbed virus was removed by acid washing cells with MES-buffered saline (25 mM morpholineethanesulfonic acid (MES]), 145 mM NaCl, pH 5.5) and two subsequent neutralisation washes with culture medium (DMEM/F12, with HEPES, L-Glutamine (Sigma, UK) containing 1% (v:v) fetal calf serum (FCS) and antibiotics). Supernatants were collected at 0, 2, 4, 6, 8 and 24 hours post-infection (hpi) to determine the viral titers by plaque assay. The plaque assay was performed by infecting IBRS-2 cell monolayers with serial 10-fold dilutions of FMDV virus stocks, overlaid with indubiose (MP Biomedicals, UK (Cat # 11AGAI0025) and incubated for 48 h at 37 °C. Cells were fixed and stained with methylene blue staining and plaque forming units (PFU)/ml determined.

### Virus purification

Following cytopathic effect (CPE) of infected goat epithelium cells, virus within clarified supernatants was precipitated with 7.5% (w/v) PEG 6,000. Precipitated pellets were resuspended in PBS, centrifuged at 2,060 g for 20 min at 4 °C to remove debris and pelleted over 30% sucrose cushions by centrifugation at 104,000 g for 2.5 h at 12 °C. Pellets were resuspended in PBS/0.5% (v/v) IGEPAL CA-630 (Sigma Aldrich, UK), overlayed onto a 15–30% sucrose gradient and then fractionated by centrifugation at 104,000 g for 3 h at 12 °C. Virus concentrations were determined by quantifying sucrose gradient fractions spectrophotometrically and using the following formula: (OD_260_ × Total volume)/7.6) = mg of virus.

Antigen used for animal studies, and analysed by ELISA, was first PEG precipitated and pelleted over a 30% sucrose cushion as described above, before being resuspended in PBS.

### Cryo-EM data collection, structure determination and model building

Aliquots of 4 μl of purified inactivated SAT2, SAT2/O, ts-SAT2/O or SAT2/O mut2 virus were incubated on glow-discharged holey carbon-coated copper grids (C-flat, CF-2/1-2C; Protochips) for 30 s. Grids were blotted for 3 s to remove unbound sample, in 70% relative humidity, and vitrified in liquid ethane with a plunger device (Vitrobot; FEI). Images were collected at 300 kV on a Tecnai F30 ‘Polara’ (Oxford) or Titan Krios (Diamond Light Source) microscope (FEI), both equipped with an energy filter (GIF Quantum, Gatan) operating in zero-loss mode (0–20 eV energy selecting slit) and a direct electron detector (K2 Summit, Gatan). Movies (25 frames, each 0.2 s) were recorded at 0.8–2.8 μm underfocus in single-electron counting mode with SerialEM^38^ (Polara) or EPU (Krios) at a calibrated magnification of 37,027x or 47,169x thus resulting in a pixel size of 1.35 Å or 1.06 Å. For SAT2/O, data were collected using super resolution mode of K2 camera with a physical pixel size of 1.3 Å. Frames from each movie were aligned and averaged to produce drift-corrected micrographs^39^ (Motioncorr).

Structures were solved with RELION 1.3 or 1.4 (Supplementary Table 3) according to recommended gold-standard refinement procedures and icosahedral symmetry was applied^40^. Micrographs showing signs of astigmatism or significant drift were discarded and not used for further analysis. Reference-free 2D class averaging was used to discard bad particles. The particle population was further improved by 3D classification. The EM structure of native FMDV SAT2 (PDB: 5ACA)^17^ was low-pass-filtered to 50 Å and used as an initial template for 3D classification and refinement. The 5ACA model was fitted in the density map as a rigid body with UCSF Chimera^41^. The fitting was further improved with real-space refinement with COOT^42^. The model of SAT2/O was built based on this starting model with COOT^42^. Models were further improved by iterative positional and *B*-factor refinement in real space with Phenix^43^ and COOT^42^. Only coordinates were refined; the maps were kept constant. Each round of model optimization was guided by cross-correlation between the map and the model. Statistics of refinement are given in Supplementary Table 3.

### Thermofluor assay

Thermofluor assays were performed in PBS buffer (or 50 Mm Hepes/NaOH buffer at the desired pH) using an MX3005 PCR machine (Agilent Technologies, UK) as previously described^15,16^. RNA release assays were performed using 0.4 μg of virus and SYBR green-II dye (Molecular Probes, Invitrogen; final dilution 1:1000). The temperature was ramped from 25 °C to 94 °C in 0.5 °C increments with intervals of 10 s for all assays. Fluorescence was read with excitation and emission wavelengths of 490 nm and 516 nm, respectively. Data sets exported from the qPCR machine were visualized using MxPro software (Stratagene). The release of RNA and hence the dissociation of capsids (RNA release assay), was detected by increases in fluorescence signal and the melting temperatures (Tr) were taken as the minimum of the negative first-derivative of the fluorescence curve. Three independent thermofluor assays were performed for each analysis.

### Llama single-domain antibody ELISA

SAT2 antigens were incubated at different temperatures (4 °C, 40 °C or 47 °C) for 10 minutes and their stability was analyzed by double antibody sandwich ELISA using llama single-domain antibody fragments (VHH domains), termed M377F VHH, against intact FMD viral particles (146 S infectious virions). M377F VHH was provided by the Central Veterinary Institute of Wageningen UR, AB Lelystad, The Netherlands^18^. The wells of a 96-well microtitre plate were incubated overnight with VHH domains in carbonate/bicarbonate buffer (50 mM, pH 9.6; Sigma Aldrich, UK) at a concentration of 0.5 mg/l. Test samples serially diluted in VHH buffer (1% skimmed milk; 0.05% Tween; 0.5 M NaCl; 2.7 mM KCl; 2.8 mM KH_2_PO_4;_ 8.1 mM Na_2_HPO_4_, pH 7.4.) were added to the wells and incubated for 1 hour at 37 °C. Next, the biotinylated VHH-M377F, diluted in VHH buffer at a concentration of 0.1 mg/l, was added and incubated for 1 hour at 37 °C. Plates were washed with PBS-Tween (0.05% Tween 20) and bound biotinylated VHH-M170F was visualised by incubation with streptavidin-HRP (Dako, UK) for 1 hr at 37 °C followed by addition of *o*-phenylenediamine dihydrochloride (Sigma, UK) for 15 minutes at room temperature. The reaction was stopped by addition of 1.25 M sulphuric acid and quantified at 492 nm using a Dynex microplate reader (Dynex Technologies, UK). ELISAs were performed twice in triplicate.

### Vaccination of cattle with FMDV vaccines

Two inactivated FMDV antigen stocks, wild type SAT2 ETH/65/09 (v2-SAT2) and chimeric SAT2 ETH/65/09 (v2-SAT2/O), were prepared alongside each other using identical procedures. Equal quantities (20 μg/animal) of each antigen stock was incubated at 45 °C for two hours prior to overnight storage at 4 °C. The following day (day 0), two groups of five 100- to 150-kg male Holstein Friesian calves were each vaccinated with 2 ml of formulated vaccine, comprised of equal amounts (1 ml) of heated v2-SAT2 or v2-SAT2 antigen and an equal volume (1 ml) of oil adjuvant ISA201 (SEPPIC), as an intramuscular injection. Booster vaccines, prepared in the same way from the same antigens (stored at 4 °C), were administered on day 21 of the study. Control animals were vaccinated, and boosted on day 21, with 9 µg (146S) of unheated SAT2 v2-SAT2 or v2-SAT2/O antigen formulated in ISA201. All animals were bled to collect serum on days 0, 7, 14, 21 and 28, and virus-neutralizing-antibody titers (VNT) were assessed. Animal experimentation was approved by the Pirbright Institute Ethical Review Board under the authority of a Home Office project license (70/7253) in accordance with the Home Office Guidance on the Operation of the Animals (Scientific Procedures) Act 1986 and associated guidelines. All studies complied with the Council Directive 86/609/EEC on the approximation of laws, regulations and administrative provisions of the Member States regarding the protection of animals used for experimental and other scientific purposes. Group sizes for study were consistent with those in previously published studies used to determine the induction of antibody titers consistent with protection^20,44^. Animals were distributed within the groups by random permutations using an online research randomizer program (www.randomizer.org). The laboratory staff members performing the assessment of antibody titers were blind to the animal group allocations.

### Titration of neutralizing antibodies

Clarified stocks of unpurified virus were used for titration of neutralising antibodies. Serum virus neutralization antibody titers (VNTs) were performed according to the protocol recommended by the World Organisation for Animal Health (Office International des Epizooties (OIE))^45,46^. Both v2SAT2 and v2-SAT2/O viruses were tested in parallel on BHK-21 cells against homologous and heterologous sera. Sera were inactivated at 56 °C for 30 min before use. Neat serum stocks were initially diluted 1:8 and then in two-fold dilutions for the tests (1:8, 1:16, 1:32, 1:64, 1:128, 1:256, 1:512, 1:1024). For each test a 100 TCID50 of virus was used in a total volume of 50 μl. Neutralizing antibody titres, calculated by the Spearmann-Karber method^47^, were expressed as the last dilution of serum that neutralizes 50% of the virus. VNTs were performed twice in triplicate and yielded comparable results.

## Electronic supplementary material

Supplementary figures and tables
