# Supplementary material for: Chimeric O1K foot-and-mouth disease virus with SAT2 outer capsid as an FMD vaccine candidate
Source: Sci Rep. 2018 Sep 12;8:13654. doi: 10.1038/s41598-018-31856-x (PMC6135822; doi:10.1038/s41598-018-31856-x)
Supplement: Supplementary file 1 — Supplementary figures and tables [file 41598_2018_31856_MOESM1_ESM.pdf]

Chimeric O1K foot-and-mouth disease virus with SAT2 outer capsid as an FMD vaccine candidate

Abhay Kotecha[3†], Eva Perez-Martin[1†], Yongjie Harvey[1], Fuquan Zhang[1], Serban Ilca[3], Elizabeth E. Fry[3], Ben Jackson[1], Francois Maree[2], Katherine Scott[2], Corey W. Hecksel[5], Michiel M Harmsen[4], Valérie Mioulet[1], Britta Wood[1], Nick Juleff[1], David I. Stuart[3,5], Bryan Charleston[1] and Julian Seago[1,*]

[1] The Pirbright Institute, Woking, Surrey, GU24 0NF, United Kingdom
[2] Transboundary Animal Disease Programme, ARC-Onderstepoort Veterinary Institute, Private Bag X05, Onderstepoort 0110, South Africa.
[3] Division of Structural Biology, Wellcome Trust Centre for Human Genetics, University of Oxford, Roosevelt Drive Oxford OX3 7BN, United Kingdom.
[4] Wageningen Bioveterinary Research, Division Virology, P.O. Box 65, 8200 AB Lelystad, The Netherlands.
[5] Diamond Light Source, Harwell Science and Innovation Campus, Didcot OX11 0DE, UK.

*** Corresponding author:** Julian Seago, The Pirbright Institute, Woking, Surrey, GU24 0NF, United Kingdom. Tel: +44(0) 1483 231184; Fax: +44(0) 1483 232448; E-mail:julian.seago@pirbright.ac.uk
† Equal author contribution.

Supplementary figure 1

Cryo-EM density maps, (a) SAT2 wt (b) SAT2/O (c) SAT2/O mut 2 (d) ts-SAT2/O. Depth-cueing is used such that colour indicates radius (<110 Å: cyan; 120–140 Å: yellow; >150 Å red). (e) A snapshot of atomic model fitted into density map of ts-SAT2.

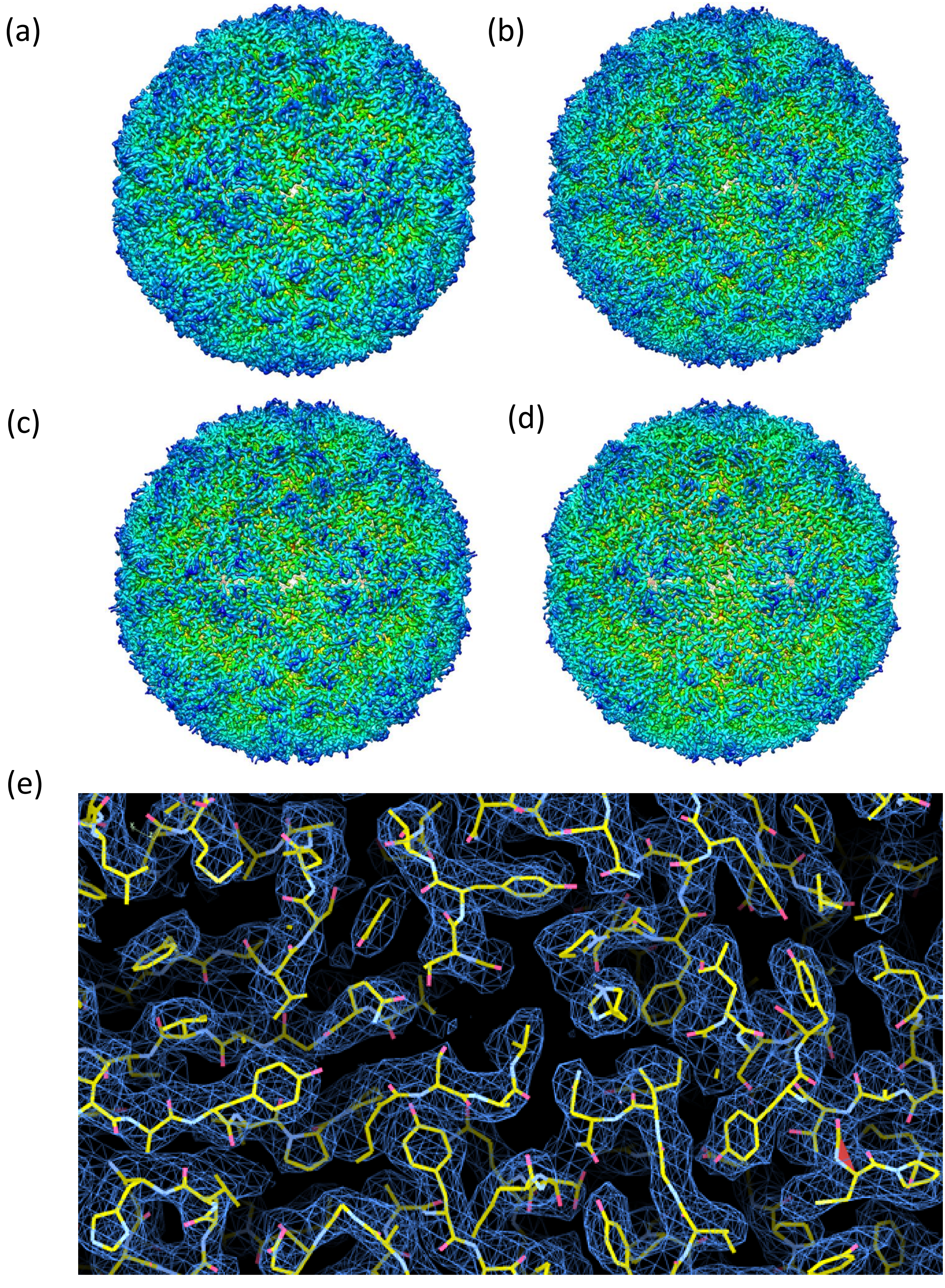

Supplementary figure 2

Disorder prediction of amino acids on VP4. The disorder predication of amino acid residues on VP4 was carried out using MoreRONN (unpublished). The residues with disorder probabilities higher than 0.5 are considered as disordered.

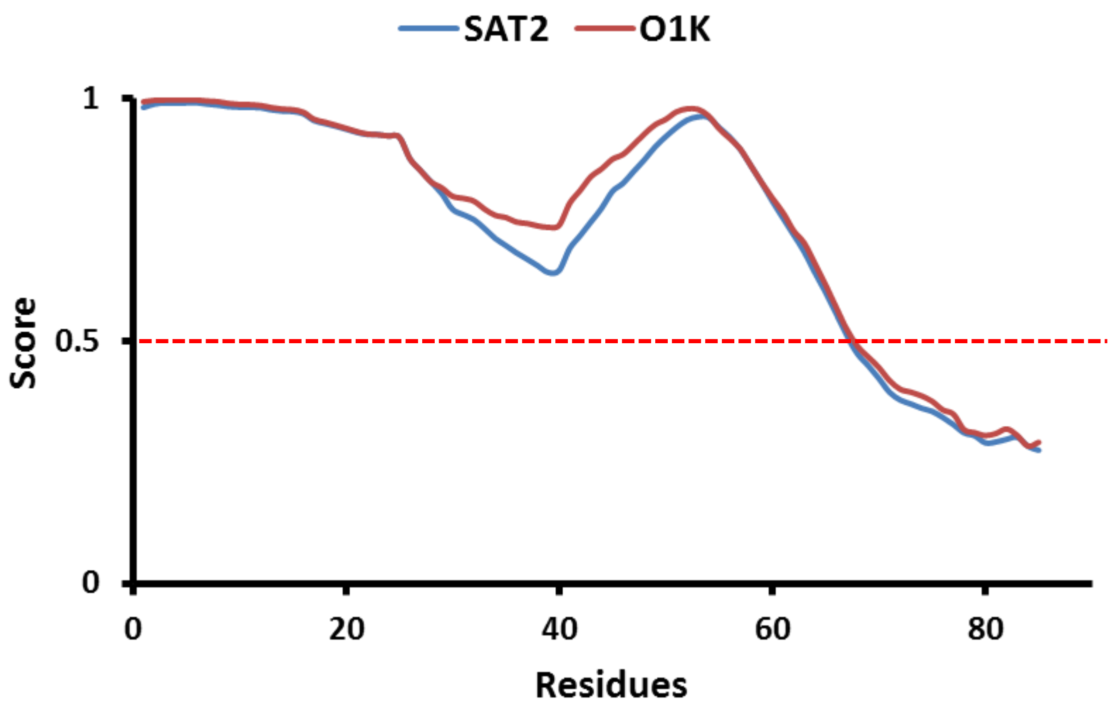

Supplementary figure 3

Polyclonal serum that detects both 146S (intact virus) and 12S (capsid subunits) was used for ELISA to confirm the presence of comparable levels of antigen before and after incubation at different temperatures. Wild type SAT2/O and SAT2/O viruses containing one (SAT2/O mut 1, SAT2/O mut 2, SAT2/O mut 3) or three targeted (SAT2/O mut 1/2/3) VP4 mutations, were incubated at 4°C, 40°C or 47°C for 10 minutes.

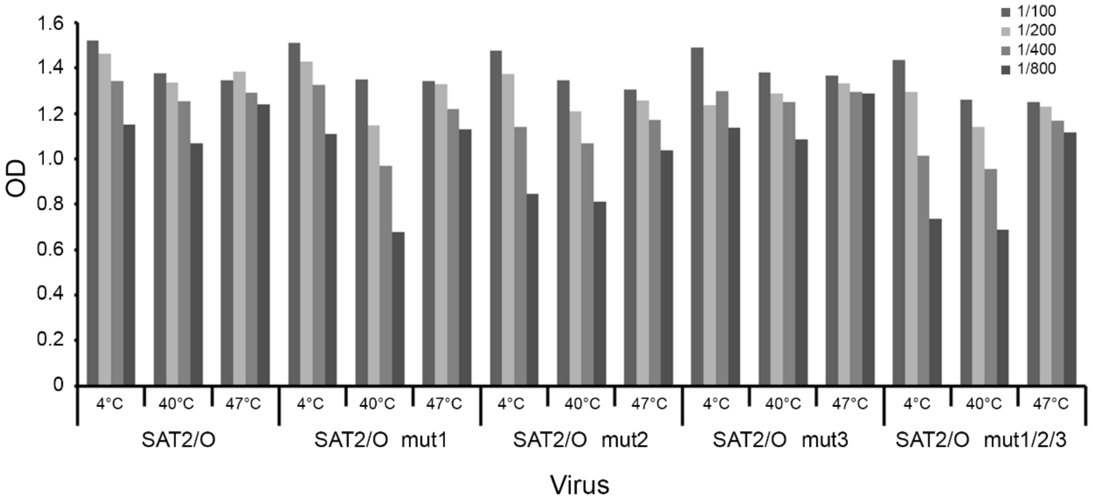

Supplementary Table 1

Wild type SAT2/O and SAT2/O viruses containing one (SAT2/O mut 1, SAT2/O mut 2, SAT2/O mut 3) or three targeted (SAT2/O mut 1/2/3) VP4 mutations, were incubated at 4°C, 40°C or 47°C for 10 minutes. Virus samples were then analysed by ELISA using a single-domain llama antibody (M377F VHH) that specifically recognises intact FMDV capsid (146S).

	SAT2/O			SAT2 Rd1			SAT2 Rd2			SAT2 Rd3			SAT2 Rd1/2/3		
Dilution	Intact 4°C	40°C	47°C	Intact 4°C	40°C	47°C	Intact 4°C	40°C	47°C	Intact 4°C	40°C	47°C	Intact 4°C	40°C	47°C
1/100	1.728	1.633	1.454	1.684	1.57	1.395	1.592	1.235	0.305	1.673	1.595	1.449	1.551	1.444	0.449
1/200	1.619	1.493	1.029	1.525	1.312	1.086	1.236	0.784	0.133	1.582	1.528	1.237	1.155	1.086	0.267
1/400	1.352	1.182	0.678	1.227	0.948	0.695	0.844	0.445	0.1	1.347	1.294	0.905	0.732	0.644	0.225
1/800	1.009	0.757	0.412	0.824	0.56	0.438	0.452	0.205	0.062	0.976	0.864	0.623	0.401	0.338	0.139

Supplementary Table 2

Thermofluor analysis to determine capsid stability of parental and chimeric SAT FMDVs at different pH values (pH 6, 6.3, 6.5, 6.8, 7.0, 8.0, 8.5 and 9.0). The respective temperatures of genome release, and hence capsid disassembly, are shown.

Virus	pH 6	pH 6.3	pH 6.5	pH 6.8	pH 7	pH 8	pH 8.5	pH 9
SAT2	28.7 ± 0.5	36.5 ± 0.3	39.3 ± 0.3	43.9 ± 0.1	46 ± 0.1	51.9 ± 0.1	51.1 ± 0.3	49.5 ± 0.0
SAT2/O	28.6 ± 0.3	36.6 ± 0.1	40.4 ± 0.3	45.5 ± 0.1	47 ± 0.1	53 ± 0.0	52 ± 0.2	50.5 ± 0.0
SAT2/O mut 1/2/3	ND	ND	41.3 ± 0.1	46.5 ± 0.0	47 ± 0.0	50.9 ± 0.1	50.9 ± 0.1	48.5 ± 0.0
SAT2/O mut 1	34.4 ± 0.1	42.7 ± 0.2	45.6 ± 0.0	48.5 ± 0.1	50 ± 0.1	53.9 ± 0.0	52.5 ± 0.0	50.9 ± 0.1
SAT2/O mut 2	26.5 ± 0.3	31.4 ± 0.3	34.8 ± 0.3	41.2 ± 0.1	42.9 ± 0.0	49 ± 0.1	48.1 ± 0.1	46.4 ± 0.4
SAT2/O mut 3	31.8 ± 0.4	40.1 ± 0.1	42.9 ± 0.0	46.5 ± 0.1	49.1 ± 0.1	52.9 ± 0.1	51.5 ± 0.0	50 ± 0.1

Supplementary Table 3

Cryo-EM data collection and refinement statistics.

Samples	SAT2	SAT2/O	SAT2/O mut 2	ts-SAT2/O
**Data Collection**				
**Microscope**	FEI Tecnai Polara	FEI Titan Krios	FEI Titan Krios	FEI Titan Krios
**Detector**	Gatan K2 Summit	Gatan K2 Summit	Gatan K2 Summit	Gatan K2 Summit
**Filter**	Gatan GIF Quantum	Gatan GIF Quantum	Gatan GIF Quantum	Gatan GIF Quantum
**Pixel size (Å)**	1.35	1.06	1.06	1.3
**Defocus range (µm)**	-2.8 to -1.0	-2.8 to -1.0	-2.8 to -0.8	-2.8 to -0.8
**Voltage (kV)**	300	300	300	300
**Electron dose (e^{-} Å^{-2})**	30	30	30	30
**Reconstruction**				
**Number of particles**	8935	6606	3554	8776
**Software**	RELION 1.3	RELION 1.4	RELION 1.4	RELION 1.4
**Symmetry**	I1	I1	I1	I1
**Final resolution (Å)**	3.5	2.8	3.7	3.6
**Map sharpening B-factor (Å^2)**	-121.7	-57.4	-122.0	-141.1
**Atomic model refinement**				
**Software**	Phenix	Phenix	Phenix	Phenix
**Resolution limit (Å)**	3.5	2.8	3.7	3.6
**Number of atoms**				
**Protein atoms**	5400	5400	5400	5207
**Other atoms**	-	-	-	-
**Residues**				
**Map CC (around atoms)**	80%	79%	77%	75%
**RMS bond lengths (Å)**	0.01	0.01	0.01	0.01
**RMS bond angles (°)**	0.94	0.96	0.83	0.97
**Validation**				
**Clashscore, all atoms (percentile)**	3.13	3.75	3.89	2.56
**Rotamer outliers (%)**	0.1	1.1	0.2	0.7
**C-beta deviations >0.25 Å**	0	0	0	0
**Ramachandran plot (%)**				
**Favored**	89.5	93.8	90.5	88.7
**Allowed**	10.4	6.1	9.3	11.0
**Outliers**	0.1	0.1	0.2	0.3
